# Cultivating Academic Writing Competence in Medical Students in the Era of Artificial Intelligence

**DOI:** 10.7759/cureus.106627

**Published:** 2026-04-08

**Authors:** Hongnan Ye

**Affiliations:** 1 Department of Research and Medical Education, Beijing Alumni Association of China Medical University, Beijing, CHN

**Keywords:** academic writing, artificial intelligence, competence, medical education, medical students

## Abstract

In an era where artificial intelligence (AI) profoundly permeates academic writing, this paper proposes a collaborative model that defines the roles of students, mentors, and AI in medical academic writing training. Its primary significance resides in mitigating the propensity of medical students to excessively depend on AI in academic writing. It deconstructs the academic writing process into three distinct phases: problem definition, literature retrieval and information synthesis, and paper drafting and iterative refinement. It clearly delineates the auxiliary boundaries for AI as an efficiency enhancer and reflection catalyst, alongside the core role of the mentor as a thought guide and value judge. The core of a successful AI-integrated education strategy lies in a meticulously designed collaborative proces.

## Editorial

Introduction

Currently, generative artificial intelligence (AI), represented by large language models, is reshaping the entire process of knowledge production. In the field of academic writing guidance, AI tools offer unprecedented convenience for literature retrieval, text summarization, language refinement, and even preliminary conceptualization of academic ideas [1]. This trend aligns with the shift in medical education from a teacher-centered approach to a modern paradigm centered on students and guided by problems. However, technological convenience carries significant educational risks: overreliance on AI may trap students in passive learning, undermining their capacity for active inquiry, deep analysis, and critical evaluation-skills that form the very foundation of academic writing. Research indicates that unguided AI use may even induce cognitive debt, weakening neural connections and diminishing overall performance [2]. Meanwhile, traditional mentorship faces challenges in scalability and immediacy, yet its provision of emotional support, experiential intuition, and personalized value guidance remains irreplaceable by AI [3]. Thus, the core question for medical educators is not whether to use AI, but how to construct an educational structure that organically integrates AI's efficiency-enabling capabilities with mentors' deep guidance to jointly cultivate students' higher-order thinking skills. Existing explorations predominantly focus on evaluating the effectiveness of specific AI tools within isolated segments of academic writing guidance, lacking a systematic and transferable pedagogical framework. This paper aims to fill this gap by proposing a trinity-based academic writing development framework: students-mentors-AI. The core concept of this framework is to view the academic writing process as a phased, iterative cognitive training program. At each stage, it clearly defines the student's primary responsibility, the boundaries of AI assistance, and the intervention points for mentors. This ensures technology becomes wings for expanding thought rather than a wheelchair for intellectual laziness.

Academic writing development framework

The framework is built upon three mutually reinforcing core principles.

Student Agency Principle

This posits that students are active constructors of their learning journey, rather than passive recipients of information. Framework design must safeguard students' full autonomy in core cognitive stages (e.g., problem formulation, argument construction) [4].

AI Augmentation Principle

AI should be positioned as a cognitive enhancement tool. Its value lies in handling highly repetitive, computationally intensive tasks (e.g., generating preliminary search strategies or performing syntax checks) or providing diverse feedback perspectives [5]. This frees cognitive resources for both students and instructors to focus on more creative and complex work.

Mentor Guidance Principle

Mentors should transition from knowledge authorities to cognitive coaches. Their core responsibility lies in prompting reflection and deepening students' thought processes through guided questioning [5]. They must also serve as the final gatekeepers for the reliability and ethical integrity of AI-generated content, thereby transmitting the values and norms of the academic community.

This framework deconstructs academic writing (using reviews as an example) into three stages: problem definition and objective formulation, literature retrieval and information synthesis, and paper drafting and iterative refinement.

Stage 1: Problem Definition and Objective Formulation - Defending Intellectual Sovereignty

This stage constitutes the intellectual bedrock of academic writing, aiming to cultivate students' ability to identify and define genuine research questions.

Students' core tasks: Upon receiving broad themes, student groups must engage in in-depth discussions without AI intervention. Drawing upon existing knowledge and interests, they should brainstorm preliminary research questions and objectives. This process compels conceptual clarification and logical deduction, serving as crucial metacognitive training.

AI assistance boundaries: During this stage, the direct use of AI to generate research questions or objectives must be explicitly prohibited. Premature AI intervention short-circuits students' creative thought processes, causing the initiation of academic writing that is not based on students' curiosity and exploration of real scientific issues, but merely on the probabilistic generation of AI.

Core role of the mentors: The mentor's focus lies not in evaluating the quality of objectives, but in guiding students through progressive questioning (e.g., ‘Why is this question significant?’, ‘What specific aspects does the what you wish to address encompass?’, ‘What is the logical relationship between these objectives?’). This prompts students to identify ambiguities in their definitions and logical discontinuities, thereby autonomously refining broad interests into clear, specific, and actionable writing objectives. The mentor's involvement ensures the purity and depth of this cognitive training.

Stage 2: Literature Review and Information Synthesis-Empowering Efficient Inquiry

The objective of this stage is to train students to efficiently and accurately acquire and preliminarily process information.

Students' core tasks: Independently design preliminary literature search strategies incorporating keywords and Boolean operators based on defined objectives. Subsequently, conduct a critical reading of selected literature and draft preliminary summaries.

The enhanced value of AI: AI can deliver significant benefits at this stage. Students can input their self-designed search strategies into AI and request it to evaluate and optimize these strategies as a medical information expert. The key lies in students comparing and analyzing the differences between the pre- and post-optimization strategies, understanding the underlying logic, thereby making implicit retrieval techniques explicit and achieving a substantial enhancement in information literacy. After completing the literature summary, students can submit it to the AI, requesting it to provide feedback on clarity, accuracy, and relevance as a peer researcher to serve as a reference for self-revision.

Core role of the mentors: Mentors should conduct spot checks on students' search strategy optimization processes and literature selection outcomes, focusing particularly on resource quality, relevance, and authority. Simultaneously, they should guide students to critically evaluate AI feedback, discerning the validity of its suggestions to prevent unthinking acceptance. This constitutes early training in academic judgement.

Stage 3: Paper Drafting and Iterative Refinement - Deepening Critique and Innovation

This stage aims to synthesise information, construct arguments, and ultimately produce text compliant with academic standards.

Students' core tasks: Integrate personal summaries to collaboratively draft the review paper; undertake multiple rounds of revision based on feedback; and conduct a final reflection on the learning process, particularly a critical analysis of AI tool utilization.

AI simulation and support: Upon completing the first draft, submit it to AI with the instruction: Simulate an academic journal reviewer and provide constructive feedback on structural integrity, logical reasoning, content coherence, and adherence to academic standards. AI can swiftly identify superficial and structural textual issues while offering diverse perspectives for revision. Additionally, AI may be employed for later-stage linguistic refinement to ensure professional and fluent expression.

Core role of the mentors: With foundational and technical issues addressed in preceding stages, the mentor's feedback can transcend linguistic and structural concerns to focus on higher-order dimensions: Is the depth of argumentation sufficient? Does the synthesis and comparison of diverse research findings demonstrate critical engagement? Are conclusions insightful or innovative? Have academic ethics (e.g., citation standards) been rigorously upheld? At this stage, the mentor acts as both academic gatekeeper and intellectual catalyst, elevating the student's work from a correct summary to an insightful discourse.

Challenges and future direction

It must be acknowledged that implementing this framework presents multiple challenges. Firstly, it requires mentors to develop new competencies: not only must they possess subject expertise, but they must also understand the potential and limitations of AI tools, and master methods for effective questioning and guidance within an AI-assisted context. Secondly, students may harbor reluctance towards deep thinking, stemming from long-standing habits of passive learning. Finally, institutions must commit resources to developing assessment tools that evaluate not only final outputs but also the critical thinking, information literacy, and ethical awareness demonstrated by students throughout collaborative processes. Future research should focus on empirically testing this framework's efficacy, comparing its outcomes with traditional models or alternative AI integration approaches in terms of cultivating students' long-term academic capabilities, critical thinking dispositions, and ethical decision-making proficiency. Concurrently, exploring the development of AI-assisted tools specifically designed for educational contexts - offering greater transparency and interpretability - will represent a significant technological advancement direction.

The 'student-mentor-AI' academic writing framework proposed in this article provides medical educators with a roadmap for reconstructing academic writing training in the era of AI. By purposefully deploying AI in areas that enhance rather than weaken cognitive processes, and always focusing on mentor guidance and student active construction, we can transform technological challenges into educational opportunities. Ultimately, our goal is not only to teach students to write a well-qualified academic paper, but also to cultivate in them a lifelong core ability to maintain independent thinking amidst the prevalence of technology. This will be the most valuable quality for future medical scientists.
